# Henry H. Burchard, M.D., D.D.S.

**Published:** 1900-08

**Authors:** 


### Obituary.

### HENRY H. BURCHARD, M.D., D.D.S.

Dr. Burchard died June 25, 1900, at Redlands, Cal.

This announcement by telegraph, though long anticipated, deeply saddened a large circle of friends in his native city. It has been difficult to become reconciled to the early death of this brilliant writer and teacher.

Dr. Burchard was born in Philadelphia, September 20, 1862, and finished his preliminary education at the Philadelphia High School. In 1879 he entered the engineer class of the United States navy, but left that to take up the study of dentistry.

He began his practical studies in this profession in 1881, and in 1884 opened his own mechanical laboratory in connection with Dr. Robert Nones. In 1885 he graduated as Doctor of Dental Surgery (D.D.S.) at the Philadelphia Dental College. In 1888 he graduated from the Jefferson Medical College of Philadelphia and began the practice of medicine, which he abandoned after three years' experience and returned to the practice of dentistry, which he continued until failing health obliged him to resort to teaching and literary work.

He was made Demonstrator of Anatomy in the Philadelphia Dental College in 1886.

His contributions to dental periodical literature have been varied and important. In 1896 he was elected to the Chair of Pathology and Therapeutics in the Philadelphia Dental College, and continued in this service until obliged to resort to a more congenial climate.

The principal work of his later years, and which contributed largely to the further undermining of an already enfeebled organization, was the editing and the contribution of eight chapters to Essig's "Prosthetic Dentistry." He also wrote several chapters and made one hundred drawings for Kirk's "Operative Dentistry." This laborious work was immediately followed by the preparation of his own book of "Dental Pathology, Therapeutics, and Pharmacology," issued in 1898. He had previously prepared a "Compend on Dental Pathology and Therapeutics." In addition to the before-mentioned works, he revised the dental definitions for Duane's Dictionary, and also performed the same service for Gould's

Dictionary. He wrote sections on Plastics, Vol. IV., "American System of Dentistry," and revised dental portions in the thirteenth edition of Gray's "Anatomy," besides contributing extensively to the illustrations. He was also one of the assistant editors in the science department of Catching's "Compendium." He was a member of several dental societies, also of the Franklin Institute of Philadelphia and the Biological Section of the Academy of Natural Sciences of the same city.

This brief *résumé* of his work furnishes but a limited idea of his untiring industry. He seemed to feel that he must not only work while the day lasted, but far into the nights, and, while he enjoyed social life, he allowed himself but little time for its pleasures.

While he lived only thirty-eight years, he accomplished much more than most men of three score years and ten.

Dr. Burchard was a student of others' work rather than an original investigator; indeed, as he acknowledged to the writer, he had little taste or ability in this direction. His power, however, to grasp the thought of his time and make it his own exceeded that of any one the writer ever knew. Gifted with a remarkable memory, nothing escaped him and, apparently, nothing was lost. Coupled with this he possessed a brilliant force in the use of language, and could hold the attention of his hearers whether in scientific societies or before his classes. The result was that he became a popular teacher. It was pathetic, when suddenly paralyzed while reading in the midst of his family, to witness the efforts of this brilliant mind to find words to express his thoughts.

This was the beginning of the end, and shortly thereafter he removed to Redlands, Cal., and there ended his career and was buried at Hillside Cemetery in that place.

The writer feels that this departure from the active work of earth of one so young and so gifted is something more than the passing on of the ordinary man. He died, as probably he would have desired, full of energy and work to the last, but this life and its results seem to convey a lesson in that it shows there are hours for work and hours for recreation. The mind as well as the body must have periods of activity and rest, but excess in either direction tends to weakness and eventual destruction.

Dr. Burchard leaves no successor in his native city,—one who can take up his work exactly as he left it. The place he filled in the hearts of those who loved and admired him cannot be filled, but the experiences of this life will remain a memory and an inspiration for us all to labor for his and our profession that the light which he sought to infuse into untrained minds may continue to impart its brilliancy into a larger circle, that the needs of the dental profession may be met and its problems eventually conquered.

Dr. Burchard married Miss Esther Vinson, of Philadelphia, April 30, 1888. She and two young daughters survive him.

